# The Anti-(+)-Methamphetamine Monoclonal Antibody mAb7F9 Attenuates Acute (+)-Methamphetamine Effects on Intracranial Self-Stimulation in Rats

**DOI:** 10.1371/journal.pone.0118787

**Published:** 2015-03-05

**Authors:** Andrew C. Harris, Mark G. LeSage, David Shelley, Jennifer L. Perry, Paul R. Pentel, S. Michael Owens

**Affiliations:** 1 Minneapolis Medical Research Foundation, Minneapolis, Minnesota, United States of America; 2 Department of Medicine, University of Minnesota Medical School, Minneapolis, Minnesota, United States of America; 3 Department of Pharmacology and Toxicology, College of Medicine, University of Arkansas for Medical Sciences, Little Rock, Arkansas, United States of America; The Scripps Research Institute, UNITED STATES

## Abstract

Passive immunization with monoclonal antibodies (mAbs) against (+)-methamphetamine (METH) is being evaluated for the treatment of METH addiction. A human/mouse chimeric form of the murine anti-METH mAb7F9 has entered clinical trials. This study examined the effects of murine mAb7F9 on certain addiction-related behavioral effects of METH in rats as measured using intracranial self-stimulation (ICSS). Initial studies indicated that acute METH (0.1-0.56 mg/kg, s.c.) lowered the minimal (threshold) stimulation intensity that maintained ICSS. METH (0.3 mg/kg, s.c.) also blocked elevations in ICSS thresholds (anhedonia-like behavior) during spontaneous withdrawal from a chronic METH infusion (10 mg/kg/day x 7 days). In studies examining effects of i.v. pretreatment with mAb7F9 (at 30, 100, or 200 mg/kg), 200 mg/kg blocked the ability of an initial injection of METH (0.3 mg/kg, s.c.) to reduce baseline ICSS thresholds, but was less capable of attenuating the effect of subsequent daily injections of METH. MAb7F9 (200 mg/kg) also produced a small but significant reduction in the ability of METH (0.3 mg/kg, s.c.) to reverse METH withdrawal-induced elevations in ICSS thresholds. These studies demonstrate that mAb7F9 can partially attenuate some addiction-related effects of acute METH in an ICSS model, and provide some support for the therapeutic potential of mAb7F9 for the treatment of METH addiction.

## Introduction

(+)-Methamphetamine (METH) addiction is a major public health problem throughout the world [1–3]. There are currently no approved pharmacotherapies for treatment of METH addiction. To date, medication development for METH addiction has focused on the use of small molecule pharmacotherapies (*e*.*g*., selective serotonin reuptake inhibitors, dopamine agonists) that target the same CNS processes that mediate METH’s addictive effects [1, 4]. However, because these same processes also mediate a variety of other functions (*e*.*g*., affect, cognition), these medications can have unwanted side effects at therapeutic doses [3].

Passive immunization with high affinity anti-METH monoclonal antibodies (mAbs) represents an alternative approach for the treatment of METH addiction [5–8]. In contrast to small molecule pharmacotherapies, anti-METH mAbs work peripherally by binding and sequestering METH in serum and reducing METH distribution to brain. This approach has several advantages for treating METH addiction. For example, anti-METH mAbs are selective for METH itself and do not bind endogenous neurotransmitters or interact with their receptors, thus avoiding CNS side effects. In addition, the unique mechanism(s) of action of anti-METH mAbs against METH (*i*.*e*., pharmacokinetic antagonism) and lack of cross-reactivity against other medications allows for its combination with other pharmacotherapies without increasing the likelihood of side effects [5–7, 9].

In the process of discovery of a candidate anti-METH mAb, several prototype mouse anti-METH mAbs were shown to reduce METH distribution to the brain and attenuate METH’s behavioral effects in rats [10–14]. Some of the key characteristics of mouse anti-METH mAb7F9 that led to its selection over other anti-METH mAbs for conversion to a human therapeutic agent (*i*.*e*., chimeric anti-METH mAb7F9, ch-mAb7F9) were its high affinity for METH (*K*_D_ = 7.7 nM), lack of cross-reactivity with structurally related drugs and neurotransmitters, a long functional half-life, and a strong safety profile [6, 9, 15, 16].

Consequently, Phase 1 clinical trials have begun to assess the safety and efficacy of ch-mAb7F9 for the treatment of METH addiction (ClinicalTrials.gov Identifier: NCT01212185). Despite the advancement of ch-mAb7F9 to clinical trials, continued preclinical evaluation of murine mAb7F9 in addiction-related behavioral models is essential to further the iterative development process of the most effective mAb-based therapies. However, apart from studies showing that mAb7F9 attenuates the locomotor stimulant effects of METH in rats [16, 17], the effects of mAb7F9 have not been reported in other animal behavioral models. Toward this end, the present study evaluated the ability of mAb7F9 to block METH’s effects in an intracranial self-stimulation (ICSS) model.

ICSS has been used extensively to study the behavioral effects of addictive drugs. Acute injection of a variety of drugs of abuse lowers the minimal (threshold) stimulation intensity that maintains ICSS [18, 19, 20], reflecting an enhancement of the reinforcing effects of the brain stimulation. This effect may reflect the more general ability of drugs to enhance the reinforcing effects of environmental stimuli (*e*.*g*., sensory stimuli, food) [21–24], a phenomenon receiving increasing attention as an important behavioral mechanism mediating drug addiction [23–25]. Conversely, cessation of chronic exposure to numerous drugs of abuse (*e*.*g*., amphetamine, nicotine) results in elevated ICSS thresholds, a putative measure of the diminished sensitivity to reinforcing stimuli (anhedonia) associated with drug withdrawal [26–28]. Importantly, withdrawal-induced elevations in ICSS thresholds can be reversed by administration of the drug used to induce dependence [29]. ICSS therefore appears to represent a measure of several factors thought to contribute to drug addiction. These factors include reinforcement-enhancement, withdrawal, and its relief by additional drug exposure.

The goal of this study was to examine whether mAb7F9 could attenuate the effects of acute METH on ICSS in rats. Because METH effects on ICSS have not been evaluated extensively using the discrete-trial current-threshold procedure employed in these studies, we initially established procedures for measuring 1) reductions in baseline ICSS thresholds following acute METH injection, and 2) elevations in ICSS thresholds during withdrawal from a chronic METH infusion, and 3) reversal of this effect by acute METH (Experiment 1). We subsequently examined the ability of mAb7F9 to block METH’s ICSS-lowering effects following acute daily injections of METH (Experiment 2). Finally, we examined whether mAb7F9 influenced the ability of acute METH to reverse elevations in ICSS thresholds during METH withdrawal (Experiment 3).

## Materials and Methods

### Animals

A total of 74 experimentally-naive, male Sprague Dawley rats (Harlan, Indianapolis, IN) were used in these studies, with separate groups of animals used in Experiments 1 (n = 18), 2 (n = 33), and 3 (n = 23). Rats weighed 275–300 g at arrival and were individually housed in a temperature- and humidity-controlled colony room with unlimited access to water. Rats were housed under a reversed 12-hour light/dark cycle and tested during the dark (active) phase. Beginning 1 week after arrival, rats were food-restricted to ≈18 g/day of rat chow to facilitate operant performance, avoid detrimental health effects of long-term ad libitum feeding, and limit catheter migration in Experiments 2 and 3. Animal husbandry and experimental protocols were approved by the Institutional Animal Care and Use Committee of the Minneapolis Medical Research Foundation in accordance with the 2011 NIH Guide for the Care and Use of Laboratory Animals and the Guidelines for the Care and Use of Mammals in Neuroscience and Behavioral Research (National Research Council 2003). No animals were euthanized as part of these studies. All efforts were made to minimize animal suffering.

### Drugs

(+)-Methamphetamine hydrochloride (METH; National Institute on Drug Abuse, Bethesda, MD) was dissolved in sterile saline. METH doses are expressed as the base. All injections were administered s.c. in a volume of 1.0 ml/kg. The anti-METH monoclonal antibody mAb7F9 was developed, produced, and purified as described previously [9, 15]. mAb7F9 is a murine igG1, *k* light chain with significant cross-reactivity for METH (*K*_D_ = 7.7 nM) and (+) 3,4-methylenedioxy-N-methylamphetamine [(+) MDMA; *K*_i_ = 7.9 nM], but not against (+) amphetamine (*K*_i_ = 370 nM) or the (-)-isomers of amphetamine-like drugs [9, 15, 17]. MAb7F9 (40 mg/ml) was administered i.v. at a rate of 1 ml/min, with the total volume administered dependent on dose (see below).

### Intracranial self-stimulation

The surgery, apparatus, and training procedure used here are described in detail elsewhere [29–31]. Briefly, animals were anesthetized with i.m. ketamine (75 mg/kg) / xylazine (7.5 mg/kg) and implanted with a bipolar stainless steel electrode in the medial forebrain bundle at the level of the lateral hypothalamus. Rats were later trained to respond for electrical brain stimulation using a modified version of the Kornetsky and Esposito [32] discrete-trial current-threshold procedure [29, 31, 33]. Each ICSS session lasted ≈ 45 min and provided two dependent variables: ICSS thresholds (a measure of brain reinforcement function) and response latencies (a measure of motor function). It is well established that effects of drugs on ICSS thresholds and response latencies can be mediated by different mechanisms [29, 33, 34].

### Experiment 1a: Effects of acute METH on baseline ICSS thresholds

Rats (N = 18) were tested in daily ICSS sessions conducted Mon-Fri until threshold values were stable (*i*.*e*., less than 10% coefficient of variation over a 5-day period and no apparent trend). To habituate animals to the injection procedure, s.c. saline was administered 20 min prior to ICSS testing for at least one session and until threshold values were stable (mean number of habituation sessions ± SEM = 1.4 ± 0.1, range = 1–3 sessions). Effects of a 20-min pretreatment with METH were subsequently determined at METH doses of 0, 0.03, 0.1, 0.3, and 0.56 mg/kg. These doses bracket the range of METH doses that elicit addiction-related behavioral effects in rats, including reductions in ICSS thresholds in rate-frequency threshold procedures [35–37]. Injections typically occurred on Tues and Fri, provided that ICSS thresholds were within baseline range on intervening days, and doses were administered in a counterbalanced order.

### Experiment 1b: Effects of acute METH on withdrawal-induced changes in ICSS thresholds

Following completion of Experiment 1a, baseline ICSS sessions continued for two weeks and until threshold values were stable. Rats were then implanted s.c. with Alzet 2ML2 osmotic minipumps (Durect, Cupertino, CA) delivering a 7-day continuous infusion of either saline (SAL) or 10 mg/kg/day METH using the general procedures described in [31, 34]. This dose of METH reliably induced METH dependence as measured by elevations in ICSS thresholds in pilot studies. Rats were tested in the ICSS procedure during days 1–4 and day 7 of the infusion, at which point minipumps were removed to elicit spontaneous withdrawal. Twenty hr later, SAL-infused rats were injected with SAL (SAL + SAL group, n = 5), while METH-infused rats were injected with either SAL (METH + SAL group, n = 6) or METH 0.3 mg/kg (METH+ METH group, n = 7). Twenty min later, all rats were tested in the ICSS procedure. Rats continued to be tested in the ICSS procedure on days 2, 3, 4, and 7 following osmotic pump removal.

### Experiment 2: Effects of mAb7F9 on METH’s acute effects on ICSS thresholds

A separate group of 33 rats was prepared and trained for ICSS in daily sessions until thresholds were stable. Rats were then implanted with i.v. catheters as described in [38, 39], and then allowed to reacquire stable ICSS thresholds. Rats were then infused i.v. with either phosphate buffered saline (PBS) (PBS + SAL and PBS + METH groups, n = 7–8 / group) or 30 mg/kg, 100 mg/kg, or 200 mg/kg mAb7F9 (30 mAb + METH, 100 mAb + METH, and 200 mAb + METH groups, respectively, n = 6 group). Twenty hr later (test day 1), rats were injected with either SAL (PBS + SAL group) or METH 0.3 mg/kg (all other groups) and, 20 min later, tested in the ICSS procedure. This dose of METH was used because it markedly reduced baseline thresholds in Experiment 1a (see below). The ratios between the number of IgG binding sites (2/IgG molecule) in the 30, 100, and 200 mg/kg doses of mAb7F9 to the number of METH molecules in a 0.3 mg/kg dose were 0.18, 0.61, and 1.22, respectively. Effects of SAL or METH 0.3 mg/kg on ICSS thresholds were determined on four consecutive days to assess the time course of mAb7F9 effects.

### Experiment 3: Effects of mAb7F9 on METH reversal of METH-induced withdrawal

A separate group of 23 rats was prepared and trained for ICSS in daily sessions until thresholds were stable. Rats were then catheterized, and allowed to reacquire stable ICSS thresholds as described in Experiment 2. All rats were then chronically infused with 10 mg/kg/day METH as described in Experiment 1b. On the 7^th^ day of the infusions, pumps were removed to elicit spontaneous withdrawal and rats were immediately infused i.v. with either PBS or 200 mg/kg mAb7F9. This dose of mAb7F9 was used because it attenuated the effects of acute METH on baseline thresholds in Experiment 2 (see Results). Twenty hr later, rats were pretreated with s.c. SAL (PBS + SAL and 200 mAb + SAL groups, n = 5–6 / group) or METH 0.3 mg/kg (PBS + METH and 200 mAb + METH groups, n = 6 / group). They were tested 20 min later in the ICSS assay.

### Statistical analyses

Intracranial self-stimulation thresholds (in μA) and response latencies (in sec) during each test session were expressed as percentage of baseline (*i*.*e*., mean of the last 5 sessions prior to the first METH test session in Experiments 1a and 2, or prior to osmotic pump implantation in Experiments 1b and 3). In general, ICSS threshold and latency data were analyzed using one- or two-way ANOVA followed by Bonferroni or Dunnett *post hoc* tests as appropriate. See results of specific experiments for further details.

## Results

### Experiment 1a: Effects of acute METH on baseline ICSS thresholds

**Fig 1:**
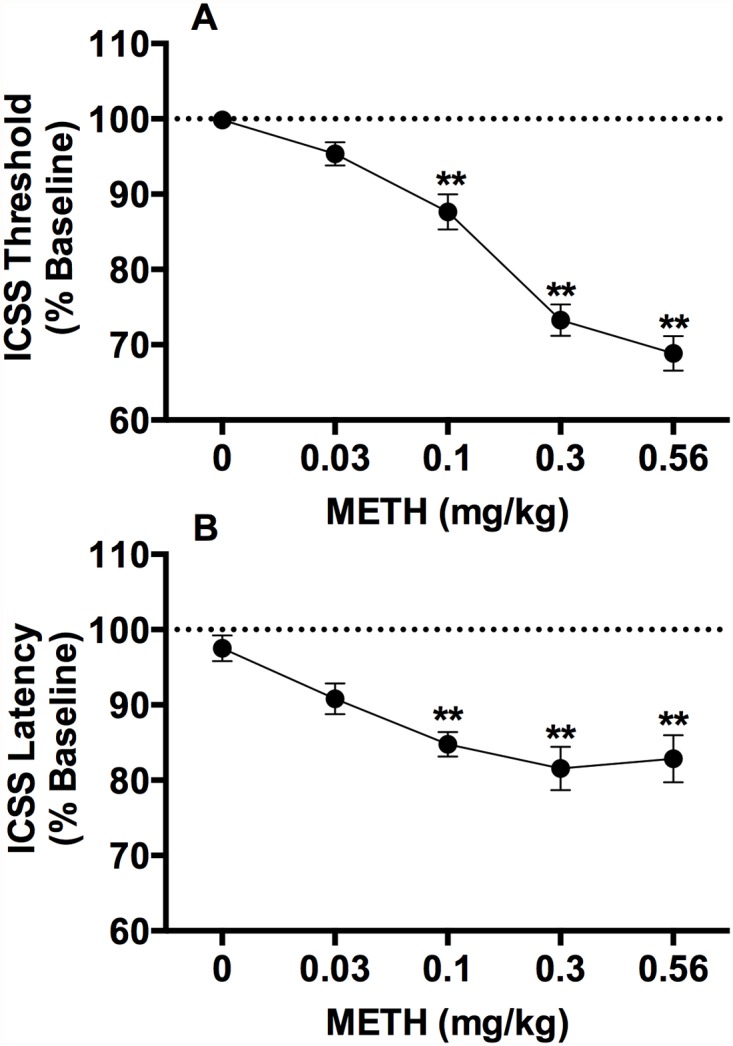
Acute injection of METH reduces ICSS thresholds. ICSS thresholds (A) and response latencies (B) (expressed as percent of baseline, mean ± SEM) following s.c. injection of METH (0–0.56 mg/kg) in Experiment 1a. ** Significantly different from 0 mg/kg METH, p < 0.01.

Baseline ICSS thresholds and response latencies were 104.6 ± 9.2 μA and 2.5 ± 0.1 sec, respectively. There were significant effects of METH dose on ICSS thresholds (*F*(4,68) = 71.0, *p* <0.0001) and response latencies (*F*(4,68) = 10.5, *p* <0.0001), with both measures reduced at the 0.1, 0.3, and 0.56 mg/kg doses compared to saline (Dunnett *q* (68) = 4.4–13.6, *p*s < 0.01: Fig. 1A and 1B).

### Experiment 1b: Effects of acute METH on withdrawal-induced changes in ICSS thresholds

**Fig 2:**
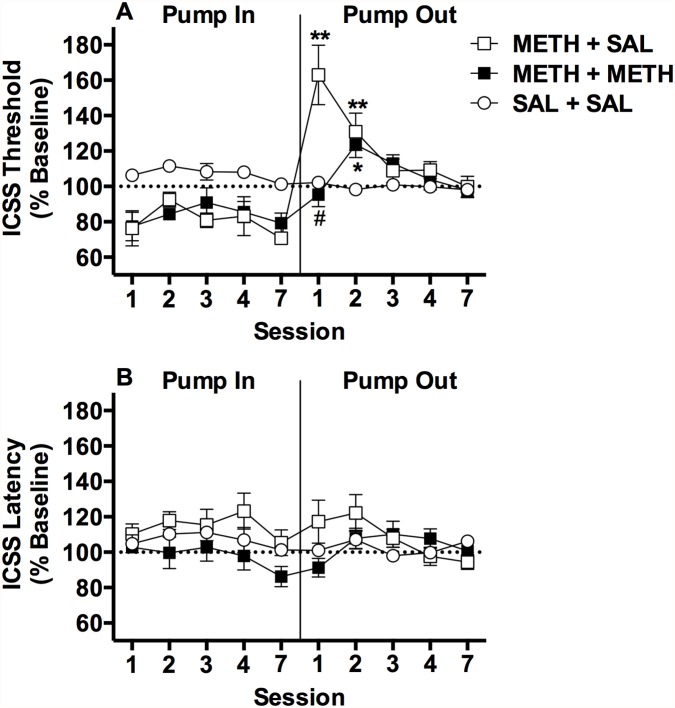
Spontaneous withdrawal from a chronic METH infusion elevates ICSS thresholds: reversal by acute METH. ICSS thresholds (A) and response latencies (B) (expressed as percent of baseline, mean ± SEM) during (“pump in” phase) and after (“pump out” phase) chronic exposure to saline or METH (10 mg/kg/day) in Experiment 1b. Rats were administered s.c. SAL or METH 0.3 mg/kg prior to the first session of the pump out phase. *^,^ ** Significantly different from SAL+ SAL at that session, p < 0.05 or 0.01. ^#^ METH + METH significantly different from METH + SAL at that session, p < 0.01. For clarity, significant differences in marginal means between the METH + SAL and METH + METH groups compared to the SAL + SAL group during the pump in phase are not shown in Fig. 2A (see text for further details).

Spontaneous withdrawal from a chronic METH infusion elevated ICSS thresholds, and this effect was reversed by acute METH (Fig. 2A). Treatment groups did not differ in terms of baseline ICSS thresholds or response latencies (Table 1). There was a significant effect of treatment group on ICSS thresholds during osmotic pump infusion (“Pump In” phase in Fig. 2A; *F*(2,15) = 15.1, *p* < 0.001), but no significant effect of session or treatment group x session interaction. Comparison of data collapsed across all test sessions during this phase (marginal means) indicated that thresholds in both METH-infused groups were reduced compared to the SAL + SAL group (Bonferroni t (15) = 4.6 or 5.0, p <0.05). There were significant main effects of treatment group (*F*(2,15) = 4.2, *p* < 0.05) and session (*F*(4,60) = 9.5, *p* < 0.0001) on ICSS thresholds following minipump removal (“Pump Out” phase in Fig. 2A), as well as a significant treatment group x session interaction (*F*(8,60) = 10.8, *p* < 0.0001). Thresholds were elevated in the METH + SAL group compared to the SAL + SAL group during the first session following pump removal (t (75) = 5.8, *p* < 0.001), reflecting spontaneous withdrawal. This effect was blocked by acute METH (Fig. 2A), as thresholds in the METH + METH group did not differ from the SAL + SAL group and were significantly lower than thresholds in the METH + SAL group (t (75) = 7.0, p < 0.001). Thresholds were elevated in both the METH + SAL and METH + METH groups compared to the SAL + SAL group on the second day of withdrawal (t (75) = 2.5 or 3.1, p < 0.05 or 0.01). No other significant between-group differences were observed during the remaining sessions (Fig. 2A).

**Table 1:** Mean (±SEM) ICSS thresholds (in μA) and response latencies (in sec) in experimental groups during baseline sessions in Experiments 1b, 2, and 3.

	Thresholds	Latencies
**Experiment 1b**		
SAL + SAL	127.8 ± 11.5	2.5 ± 0.1
METH + SAL	103.3 ± 16.8	2.2 ± 0.1
METH + METH	98.2 ± 14.9	2.3 ± 0.2
**Experiment 2**		
PBS + SAL	131.2 ± 23.0	2.9 ± 0.3
PBS + METH	110.9 ± 17.4	2.6 ± 0.3
30 mAb + METH	131.8 ± 26.0	2.6 ± 0.2
100 mAb + METH	113.1 ± 19.1	2.7 ± 0.1
200 mAb + METH	114.4 ± 19.1	2.9 ± 0.2
**Experiment 3**		
PBS + SAL	141.7 ± 29.7	2.9 ± 0.1
PBS + METH	141.5 ± 19.6	2.9 ± 0.2
200 mAb + SAL	131.5 ± 31.2	2.7 ± 0.1
200 mAb + METH	124.9 ± 14.0	2.6 ± 0.2

There was a significant main effect of session on response latencies during minipump exposure (*F*(4,60) = 3.4, *p* < 0.05), but no significant effect of group or group x session interaction (Fig. 2B). There was no significant effect of group on response latencies following pump removal, but there was a significant effect of session (*F*(4,60) = 2.7, *p* < 0.05) and a group x session interaction (*F*(8,60) = 3.3, *p* < 0.01). Latencies in the METH + METH group tended to be reduced compared to the METH + SAL group on the first withdrawal day (t (75) = 3.0, p = 0.06; Fig. 2B). No other between-group differences were observed.

### Experiment 2: Effects of mAb7F9 on METH’s acute effects on ICSS thresholds

**Fig 3:**
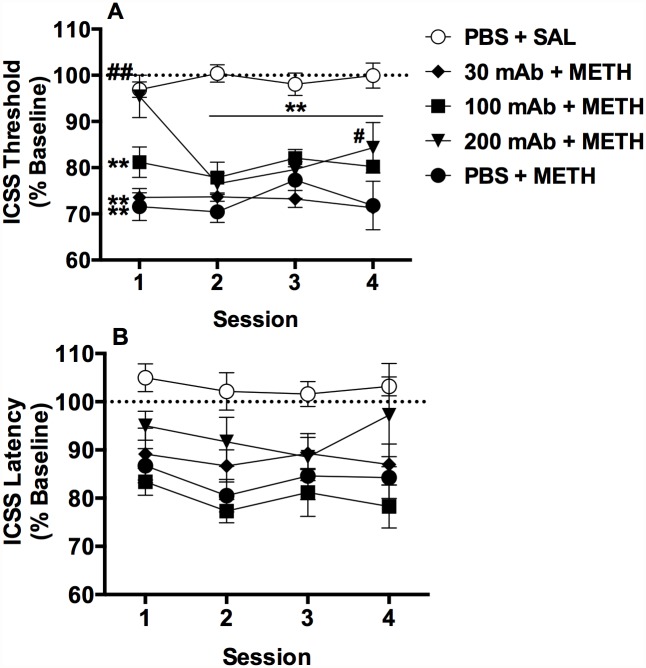
MAb7F9 attenuates the ability of acute METH to reduce baseline ICSS thresholds, ICSS thresholds (A) and response latencies (B) (expressed as percent of baseline, mean ± SEM) following i.v. infusion of vehicle (PBS) or mAb (30, 100, or 200 mg/kg) followed by repeated daily injections of s.c. SAL or 0.3 mg/kg METH in Experiment 2. ** Significantly different from PBS + SAL at that session, p < 0.01. ^#, ##^ 200 mAb + METH significantly different from PBS + METH at that session, p < 0.05 or 0.01. For clarity, significant differences in marginal means between groups are not shown in Fig. 3A or 3B.

Baseline ICSS thresholds and response latencies did not differ between treatment groups (Table 1). There was no significant effect of session on ICSS thresholds, but there was a significant effect of treatment group (*F*(4,28) = 32.7, *p* < 0.0001) and a significant treatment group x session interaction (*F*(12,84) = 2.2, *p* < 0.05). Acute METH reduced thresholds in the PBS + METH group compared to the PBS + SAL group, and magnitude of this effect remaining constant across all four test sessions (t (112) = 5.1–7.3, ps < 0.01; Fig. 3A). This effect was blocked by 200 mg/kg mAb during the first test session, as thresholds in the 200 mAb + METH group did not differ from the PBS + SAL group and were significantly elevated compared to the PBS + METH group during session 1 (t (112) = 5.6, p < 0.0001). 200 mg/kg mAb was less effective on subsequent test days, as thresholds in the 200 mAb + METH group were significantly reduced compared to PBS + SAL during sessions 2–4 (t (112) = 3.5–5.4, *p*s < 0.01) and did not differ from the PBS + METH group during sessions two and three. However, there was a small but significant difference between the 200 mAb + METH and PBS + METH groups during session four (t (112) = 2.9, p < 0.05), suggesting some efficacy of 200 mg/kg mAb on the final test day. Thresholds in the 30 mAb + METH and 100 mAb + METH groups were significantly reduced compared to PBS + SAL on all test days (t (112) = 3.6–5.4, ps < 0.01), and did not differ from PBS + METH on any individual test day (Fig. 3A). Nevertheless, comparison of data collapsed across all four test sessions (marginal means) indicated that the 100 mAb + METH group differed from the PBS + METH group (t (28) = 2.8, p <0.05), suggesting a modest attenuation of METH’s effects by 100 mg/kg mAb throughout the protocol.

There was a significant effect of treatment group on response latencies (*F* (4,84) = 6.2, *p* < 0.0001), but no significant effect of session or treatment group x session interaction (Fig. 3B). Comparison of marginal means indicated that latencies in all groups, except the 200 mAb + METH group, were reduced compared to the PBS + SAL group (t (28) = 2.9–4.4, ps < 0.05 or 0.01), indicating that 200 mg/kg mAb disrupted METH’s locomotor stimulant effects as measured by reductions in response latencies.

### Experiment 3: Effects of mAb7F9 on METH reversal of METH-induced withdrawal

**Fig 4:**
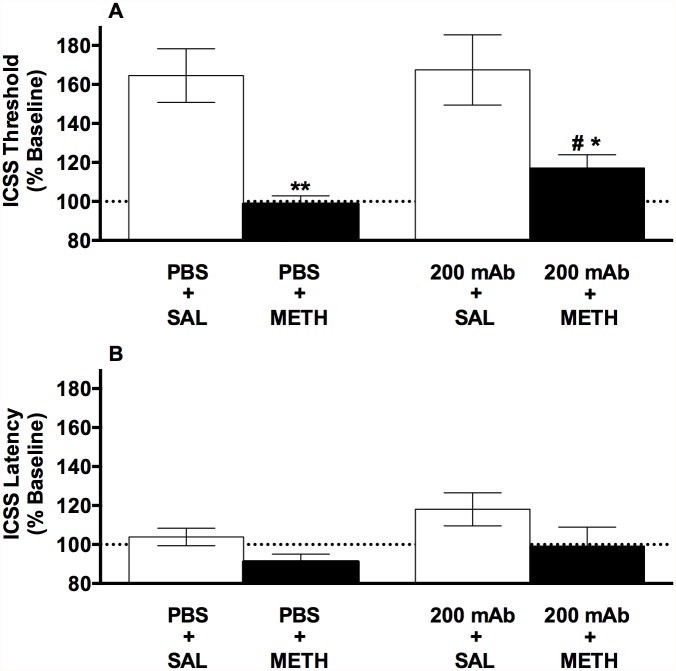
MAb7F9 modestly attenuates the ability of acute METH to reverse elevations in ICSS thresholds during METH withdrawal. ICSS thresholds (A) and response latencies (B) (expressed as percent of baseline, mean ± SEM) in rats administered i.v. PBS or 200 mg/kg mAb immediately following cessation of a chronic METH infusion, followed 20 hr later by s.c. SAL or 0.3 mg/kg METH in Experiment 3. *^,^ ** Significantly different from SAL-injected animals for that mAb dose, p < 0.05 or 0.01. ^#,^mAb 200 + METH significantly different from PBS + METH, p < 0.05.

Treatment groups had similar baseline ICSS thresholds and response latencies (Table 1). There was a significant effect of acute METH dose (0 or 0.3 mg/kg) on ICSS thresholds on the withdrawal day (*F*(1,19) = 26.1, *p* < 0.0001), but no significant effect of mAb dose (0 or 200 mg/kg) or METH dose x mAb dose interaction. Thresholds in both the PBS + SAL and 200 mAb + SAL groups were elevated compared to baseline (Fig. 4A). METH attenuated this spontaneous withdrawal effect regardless of mAb dose, as thresholds in both the PBS + METH and 200 mAb + METH groups were reduced compared to their respective SAL-injected controls (t (19) = 4.2 or 3.1, p < 0.01 or 0.05) (Fig. 4A). Nevertheless, thresholds in the 200 mAb + METH group were slightly but significantly higher than those in the PBS + METH group (t(10) = 2.3, p < 0.05), suggesting that 200 mg/kg mAb modestly attenuated the effects of METH in this protocol.

There was a significant effect of METH dose on ICSS latencies on the first day of withdrawal (*F*(1,19) = 5.1, *p* < 0.05), but no significant effect of mAb dose or interaction. Latencies in both METH-injected groups tended to be lower than their respective SAL-injected controls (Fig. 4B), although these effects were not significant.

## Discussion

This study reports the effects of the high affinity murine anti-METH mAb7F9 in a preclinical rat model of METH addiction. Experiment 1 established behavioral models for measuring the ability of acute METH to reduce baseline ICSS thresholds and reverse withdrawal from a chronic METH infusion. In Experiment 2, mAb7F9 (200 mg/kg) blocked the ICSS threshold-lowering (*i*.*e*., reinforcement-enhancing) effects of a single METH injection, but was less effective in attenuating the effects of subsequent daily METH injections. MAb7F9 at 100 mg/kg produced a small but significant attenuation of METH’s ICSS threshold-lowering effects across all test sessions, while 30 mg/kg mAb7F9 was ineffective in this model. In Experiment 3, 200 mg/kg mAb7F9 modestly attenuated the ability of acute METH to reverse elevations in ICSS thresholds during METH withdrawal, although substantial reversal of withdrawal-induced elevations by METH was still evident in mAb7F9-treated rats.

The demonstration that acute METH reduces baseline ICSS thresholds in a discrete-trial ICSS threshold procedure extends previous reports of this effect using a rate-frequency ICSS procedure [35, 36]. Our study is the first to show that spontaneous withdrawal from chronic, experimenter-administered METH elevates ICSS thresholds, and reversal of this effect by acute METH administration. These data complement reports that ICSS thresholds are elevated during antagonist-precipitated withdrawal from a chronic METH infusion [40] and spontaneous withdrawal from extended access i.v. METH self-administration [41]. These effects of acute and chronic METH on ICSS thresholds are generally consistent with those observed for amphetamine and other drugs of abuse [27, 29, 42].

The ability of mAb7F9 to attenuate METH’s effects on ICSS thresholds complements two recent reports that mAb7F9 attenuated METH’s locomotor stimulant effects [16, 17], as well as reports that other murine anti-METH mAbs diminish METH’s reinforcing, locomotor stimulant, and discriminative stimulus effects in animals [10, 11, 13]. Evidence of murine mAb7F9 efficacy in rats also suggest that ch-mAb7F9 (a form that is potentially suitable for use in humans) could have therapeutic benefits for the treatment of METH addiction, although human studies are clearly needed to evaluate this possibility.

It is unclear why mAb7F9 (200 mg/kg) was effective in the ICSS assay during the first day of a METH challenge dose, but not during subsequent METH challenges. The loss of efficacy was not likely due to changes in the baseline behavioral effects of METH across daily repeated exposures (*e*.*g*., sensitization), as these effects were stable in control animals (see PBS + METH group in Fig. 3). It is also unlikely that these findings reflect rapid elimination of mAb7F9, as a substantial amount (78%) of mAb7F9 was still present in the blood stream at the start of Session 2. This calculation of the remaining mAb7F9 dose is based on the assumption of an approximately 7 day half-life for a murine anti-METH mAb in rats [43], and the fact that one mAb7F9 dose was administered 20 hrs prior to Session 1 (*i*.*e*., 44 hrs prior to Session 2).

Loss of mAb7F9 efficacy in the ICSS assay over time could reflect residual occupancy of mAb7F9 by the initial dose of METH. However, when a 1 mg/kg dose of METH is administered to rats in the presence of a 150 mg/kg dose of mAb7F9, the METH half-life is 3.6 hrs, as compared to a 1.1 hr METH half-life without mAb7F9 treatment [9]. These data suggest that METH binding to mAb7F9, which likely has a 7 day half-life, is not irreversible and that mAb7F9 can apparently completely regenerate its METH binding capacity over a 24 hr period (about seven METH half-lives). None of the above explanations therefore readily accounts for our data. Since we only studied a single dose of mAb7F9 rather than the repeated doses required to achieve steady-state levels (which is how mAb7F9 would used in humans), we cannot draw further conclusion about the clinical relevance of this finding.

In contrast to the current findings, chronically administered mAb7F9 provided continuous reduction in METH’s pharmacological effects (*i*.*e*., significant reductions in METH-induced locomotor activity and METH brain concentrations) over a one month period [16]. Methodological differences across studies that could account for this discrepancy include dependent measure, dose and route of administration of METH, and dose and number of administrations of mAb7F9. Evaluating factors contributing to the duration of efficacy of mAb7F9 on these and other behavioral effects of METH (*e*.*g*., i.v. self-administration) represents an important area for further study.

MAb7F9 was more effective at attenuating the effects of METH on baseline ICSS thresholds compared to its effects on withdrawal-induced elevations in ICSS thresholds. These rat data suggest that mAb7F9 may have differing actions on METH-related effects in different contexts. However, only human clinical trials of ch-mAb7F9 in METH users could answer the question of which addiction-related effects would or would not benefit from mAb7F9 treatment.

This study evaluated the influence of mAb7F9 on only some of METH’s withdrawal-related effects. Future studies could evaluate the potential of mAb7F9 to precipitate withdrawal in METH-dependent animals by abruptly reducing brain METH levels, or to attenuate the development of METH dependence when administered prior to onset of chronic METH exposure. Because mAb7F9 has high affinity for METH but not for amphetamine [9, 15, 17], effects of mAb7F9 on amphetamine withdrawal could be evaluated in these models as a negative control.

In conclusion, these findings suggest that anti-METH mAb7F9 can partially attenuate addiction-related behavioral effects of acute METH on ICSS thresholds in rats. Further characterization of mAb7F9 in ICSS and other preclinical models of METH addiction will help elucidate how well rat models of METH addiction can predict clinical efficacy of these biological medications.
