# Correction: The Anti-(+)-Methamphetamine Monoclonal Antibody mAb7F9 Attenuates Acute (+)-Methamphetamine Effects on Intracranial Self-Stimulation in Rats

**DOI:** 10.1371/journal.pone.0138959

**Published:** 2015-09-18

**Authors:** Andrew C. Harris, Mark G. LeSage, David Shelley, Jennifer L. Perry, Paul R. Pentel, S. Michael Owens

In the Introduction section, the ClinicalTrials.gov Identifier is incorrect in the 4^th^ paragraph of the Introduction. The correct identifier is: NCT01603147.

## References

[1] Harris AC, LeSage MG, Shelley D, Perry JL, Pentel PR, Owens SM (2015) The Anti-(+)-Methamphetamine Monoclonal Antibody mAb7F9 Attenuates Acute (+)-Methamphetamine Effects on Intracranial Self-Stimulation in Rats. PLoS ONE 10(3): e0118787 doi: 10.1371/journal.pone.0118787 25742165 10.1371/journal.pone.0118787 PMC4350938

